# Transgender and gender‐diverse identity in patients with eating disorders: A national cross‐sectional study

**DOI:** 10.1002/erv.3143

**Published:** 2024-10-19

**Authors:** Sofie M. Rasmussen, Loa Clausen, Astrid D. Højgaard, Maria L. Pop, Mikkel K. Kjeldsen, Jeanie M. Egedal, Gry Kjærsdam Telléus

**Affiliations:** ^1^ Psychiatry Aalborg University Hospital Aalborg Denmark; ^2^ Department of Clinical Medicine Faculty of Medicine Aalborg University Aalborg Denmark; ^3^ Department of Child and Adolescent Psychiatry Aarhus University Hospital Aarhus Denmark; ^4^ Department of Clinical Medicine Faculty of Health Aarhus University Aarhus Denmark; ^5^ Sexological Centre Aalborg University Hospital Aalborg Denmark; ^6^ Research Unit for Medical Endocrinology Odense University Hospital Odense Denmark; ^7^ Centre for Eating Disorders Odense University Hospital Odense Denmark; ^8^ Institute of Communication and Psychology Psychology Aalborg University Aalborg Denmark

**Keywords:** body dissatisfaction, eating disorder, gender congruence, quality of life, transgender/gender‐diverse

## Abstract

**Objective:**

This national study aimed to estimate the prevalence of the transgender and gender‐diverse identity and to assess the level of gender congruence, body dissatisfaction and quality of life among patients with eating disorders in Denmark.

**Method:**

Patients with eating disorders were compared to a matched non‐clinical comparison group. The survey included the Eating Disorder Examination Questionnaire and assessment of body dissatisfaction, gender congruence and quality of life.

**Results:**

In total 568 patients with eating disorders and 538 individuals from the non‐clinical comparison group were included. The prevalence of individuals identifying as transgender or gender‐diverse was 4.50% (*n* = 20) among patients with eating disorders, and 6.23% (*n* = 23) in the non‐clinical comparison group (*p*‐value = 0.276). The transgender and gender‐diverse individuals with eating disorders showed no statistically significant differences in eating disorder symptomatology compared to the cisgender individuals with eating disorders; however, they reported significantly more body dissatisfaction, less gender congruence and lower quality of life.

**Conclusion:**

The prevalence of transgender and gender‐diverse individuals did not differ between patients with eating disorders and the non‐clinical comparison group; however, transgender and gender‐diverse individuals with eating disorders may be characterised by pronounced body dissatisfaction and low quality of life.

## Highlights

- The prevalence of transgender and gender‐diverse individuals did not differ between patients with eating disorders and a non‐clinical comparison group.
- Transgender and gender‐diverse individuals with eating disorders showed no significant differences in eating disorder symptomatology compared to the cisgender individuals with eating disorders.
- Transgender and gender‐diverse individuals with eating disorders may represent a distinct group, being characterised by pronounced body dissatisfaction and a low quality of life.

## 1 INTRODUCTION

The literature on gender identity in patients suffering from an eating disorder (ED) is sparse, and only a few studies have reported on the prevalence of transgender and gender‐diverse individuals in clinical ED samples. The studies conducted have estimated a prevalence of transgender and gender‐diverse individuals at 6%, 7.1% and 2.2% of patients receiving treatment for EDs (Chaphekar et al., 2022; Riddle et al., 2022, 2024). Studies have found elevated ED symptomatology, measured with EDE‐Q in transgender, gender‐diverse and sexual minority individuals relative to cisgender individuals in treatment for EDs (Coelho et al., 2021; Mensinger et al., 2020). Elevated EDE‐Q scores, especially shape concern, have also been reported in both transgender and gender‐diverse individuals outside ED treatment compared to cisgender individuals (Nagata et al., 2020a, 2020b). However, the literature on the prevalence of transgender and gender‐diverse individuals in the clinical ED population remains sparse, and a clear picture is lacking.

However, the extent of existing literature focussing on EDs among transgender and gender‐diverse individuals is increasing. Research has found that both binary transgender and gender‐diverse individuals, such as genderqueer, genderfluid, or bigender face a higher risk of EDs compared to cisgender individuals (Duffy et al., 2019; Lipson et al., 2019; Nagata, Compte, et al., 2020; Rasmussen et al., 2023; Romano & Lipson, 2021; Thorne et al., 2019). A systematic review and meta‐analysis estimated the prevalence of EDs at 17.70% among transgender individuals, which is significantly higher compared to a prevalence of EDs of 1.01% in the general population (Qian et al., 2013). Furthermore, the results of the meta‐analysis showed that transgender men reported higher levels of ED than transgender women. In a study by Diemer et al. (2015), 15.85% of transgender individuals reported being diagnosed with an ED in the past year, in contrast to 1.85% of cisgender women. Studies have shown that gender congruence is an important factor associated with the risk of developing EDs. Specifically, it seems that higher congruence between one's external appearance and gender identity is associated with a lower likelihood of disordered eating symptoms, thus suggesting gender congruence as a potential protective factor regarding EDs (Barnhart et al., 2023; Roberts et al., 2021; Uniacke et al., 2021).

Body dissatisfaction is a core symptom of EDs and has been found to be pronounced in both individuals with EDs and transgender and gender‐diverse individuals (Bandini et al., 2013; Jones et al., 2016; McLean & Paxton, 2019; Meneguzzo et al., 2024; Stice & Shaw, 2002). Higher levels of body dissatisfaction are associated with lower quality of life in both individuals with EDs and transgender and gender‐diverse individuals (Lobera & Rios, 2011; Simbar et al., 2018). According to Simbar et al. (2018) a significant positive correlation was found between quality of life and body image in transgender individuals, indicating that improvement in body image will enhance the quality of life. A clinical sample of patients with EDs investigating the association between body image and quality of life found that patients with ED showed the lowest body image quality of life (first defined by Cash & Fleming, 2002) compared to a non‐clinical sample (Lobera & Ríos, 2011). Thus, body dissatisfaction and quality of life are important factors to consider concerning both patients with EDs and transgender and gender‐diverse individuals.

Whereas the literature investigating EDs and ED symptomatology in transgender and gender‐diverse individuals is growing, the research on this group in the clinical ED population remains extremely sparse (Riddle et al., 2022, 2024). As the number of individuals identifying as transgender and gender‐diverse referred to gender‐related health services continues to increase, it is essential to get a comprehensive understanding of the association between gender identity and EDs (Fielding & Bass, 2018; Hilden et al., 2021; Nolan et al., 2019). Therefore, this study will shed light on the prevalence of transgender and gender‐diverse identities, quality of life, gender congruence and body dissatisfaction in the total population of patients with EDs in Denmark.

## 2 AIM

This study aims to assess the prevalence of transgender and gender‐diverse identity in patients with EDs and to estimate the levels of gender congruence, body dissatisfaction and quality of life among patients with EDs compared to a non‐clinical comparison group.

We hypothesize that there will be a higher prevalence of individuals identifying as transgender and gender‐diverse among individuals with EDs compared to a non‐clinical comparison group.

We also wish to explore, if there is a higher prevalence of transgender men compared to transgender women among patients with an ED, and whether the ED symptomatology profile differs between transgender and gender‐diverse individuals and cisgender individuals with EDs.

## 3 METHOD

### 3.1 Study design and study population

This study was a nationwide matched cross‐sectional study based on a survey assessing gender identity, gender congruence, body dissatisfaction and quality of life in adult patients with EDs compared to a non‐clinical comparison group. The patients with ED are all individuals with a contact related to EDs in one of the public centres for EDs in Denmark. Thus, all Danish individuals receiving treatment for an ED have received this survey, but only those answering the survey were eligible for the final sample.

Three national Danish registers were used to identify and select the population, which included all adults with a contact related to a centre for EDs, as well as the non‐clinical comparison group: The Danish civil registration system (CPR) includes a 10‐digit unique personal identification number (CPR‐number) for all Danish citizens at birth or immigration (Pedersen, 2011). Furthermore, the CPR includes various personal information on, for instance name, registered sex, and date of birth. The CPR is the key variable of all personal registers. In this study, the CPR was used to define the population and enabled the sending of the survey via a secure platform (Digital Post). The Danish national patient registry (NPR) includes information from 1977 and onward on all in‐and out‐patient hospital contacts both somatic and psychiatric (Lynge et al., 2011). The NPR was used to identify the population based on the relevant contacts regarding diagnosis codes of EDs and to ensure that the non‐clinical comparison group were not registered with a contact regarding EDs. Finally, the Danish psychiatry central research registry (PCRR) includes data on all psychiatric contacts from 1969 and forward. From 1995, the PCRR was then incorporated into the NPR (Mors et al., 2011). The PCRR was used to select the non‐clinical comparison group, ensuring that they were not previously registered with an ED diagnosis.

### 3.2 Inclusion and exclusion criteria

Participants had to be registered in the CPR, residing in Denmark, and aged 18 years or older at the time of data extraction. The patients with an ED included individuals registered with at least one documented active contact of a primary diagnosis of ED according to ICD‐10 codes DF500–DF503 and DF508 in the NPR at least 30 days before data extraction. To be eligible for the non‐clinical comparison group, individuals must not have received any diagnosis of one or more EDs according to ICD‐10 codes DF500–DF503 and, DF508 and ICD‐8 code 30650 in the NPR or PCRR. Furthermore, the non‐clinical comparison group participants had to be comparable to the individuals with EDs according to the matching procedures described. Individuals not registered with a name, those registered with address protection, or individuals who were legally incapacitated were not eligible for inclusion.

### 3.3 Selection and matching procedure

All individuals with EDs had to have an active hospital contact documented in the NPR (i.e., no termination registered). For specification, the patients had to have at least one documented contact pertaining to the diagnosis codes in the NPR. The contact of the diagnosis codes had to be registered in the NPR at least 30 days before data extraction to avoid potential recent registration errors. The primary specialty of the contact of the diagnosis was classified as ‘Psychiatry’, ‘Child and adolescent psychiatry’, or ‘Endocrinology’.

Each patient with an ED was matched 1:2 with an individual from the non‐clinical comparison group per registered sex and year of birth. The matching was performed with replacement, which means that a control could be a control for multiple cases, however, cases could not be controls for other cases. In total, 1,366 patients with EDs were eligible for inclusion and matched to individuals from the non‐clinical comparison group. Of the approximately 4.5 million potential individuals for the non‐clinical comparison group, 2,729 individuals were matched to one or more patients with an ED. Three individuals from the non‐clinical comparison group were used twice in the matching process, whereas the rest were only used once.

### 3.4 Data collection

EDs in Denmark are treated in the Danish psychiatric hospital setting. The assessment procedure for EDs typically includes a diagnostic interview with the eating disorder examination (Cooper & Fairburn, 1987) or an equivalent diagnostic evaluation. Healthcare services for EDs are fully reimbursed as part of the Danish health care system.

The data collection was based on a nationwide online self‐reported survey. The survey was sent to the participants in May 2023 via a secure platform (digital post). Two reminders were sent, one after 2 weeks and the second after 5 weeks. The reminders were sent to all participants who had not responded or partially responded. The data collection lasted a total of 5 months. The survey was administered via REDCap (research electronic data capture). REDCap is an international online system developed specifically for the collection of clinical data in connection with clinical research projects (Harris et al., 2019).

### 3.5 Survey content

The survey consisted of recognized and validated instruments and additional questions developed in collaboration with researchers and clinicians from the ED and gender identity fields. The survey was pilot‐tested before distribution. Regarding the terminology of gender identity, we aimed to be careful of our wording to include all individuals. The survey included sociodemographic information, and questions related to gender identity and gender incongruence, questions about EDs and ED symptomatology, and questions about body dissatisfaction and quality of life. The following instruments were included.

#### 3.5.1 Eating disorder examination questionnaire (EDE‐Q)

The EDE‐Q is a self‐report questionnaire that measures ED behaviours and attitudes within the past 28 days (Fairburn & Beglin, 1994). It was developed from the EDE interview (Cooper & Fairburn, 1987). The EDE‐Q consists of 28‐items distributed in four subscales, including dietary restraint, eating concerns, concerns about weight, and concerns about shape, and a global score. The questions which examine the frequency and the severity of eating pathology are rated on a seven‐point Likert scale. A higher score indicates a higher degree of ED pathology (Fairburn & Beglin, 1994). The EDE‐Q has been widely used across different populations and has demonstrated good reliability and validity and to have good criterion and concurrent validity with the EDE (Mond et al., 2004). Additionally, the EDE‐Q has also been validated among Danish patients with EDs with high construct validity, but low internal structure, although EDE‐Q was still found to be a useful instrument to identify ED symptoms (Lichtenstein et al., 2021).

#### 3.5.2 Body uneasiness test (BUT)

The BUT is a multidimensional instrument assessing body uneasiness (Cuzzolaro et al., 2006). It is a unidimensional self‐report scale with 71 items divided into two scales: BUT‐A (items 1–34) measures weight phobia, body image concerns, avoidance, compulsive self‐monitoring, detachment, and estrangement feelings towards one's own body, and BUT‐B (items 1–37) assesses specific worries about particular body parts or functions. Only the BUT‐A was administered in this study. Answers are recorded with a six‐point Likert scale (0 = never, 5 = always). A higher score indicates a higher level of body uneasiness. The scale was developed and evaluated in a population of patients with EDs (Cuzzolaro et al., 2006). BUT has been validated in both patients with EDs and non‐clinical samples, showing good psychometric properties. Internal consistency was satisfactory, and the test‐retest correlation coefficients were highly significant.

#### 3.5.3 Transgender congruence scale (TCS)

The TCS measures participants' sense of congruence between their gender and body on a 12‐item scale within the prior 2 weeks (Kozee et al., 2012). The scale frames gender as “fluid” and changeable rather than binary and is, therefore, more inclusive. It is a unidimensional scale and can include both transgender and nonbinary gender identity. The scale comprises two subscales: *Appearance Congruence* (items 1–9), which reflects whether the perception of the external appearance adequately represents the gender identity, and *Gender Identity Acceptance* (items 10–12), which reflects the extent of acceptance of the gender identity. The items are rated on a five‐point Likert scale (1 = strongly disagree, 5 = strongly agree). A higher mean score indicates a greater identity congruence. The TCS has proven to have good validity and reliability (Kozee et al., 2012). The TCS has been validated in a Swedish population of transgender individuals and showed good discriminatory validity and good internal consistency (Iliadis et al., 2020). The TCS has not been validated in a Danish population, but we believe it will perform similarly due to the resemblances in Swedish and Danish culture and language.

#### 3.5.4 WHO quality of life‐BREF (WHOQOL–BREF)

The WHOQOL‐BREF is a 26‐item questionnaire measuring quality of life (WHOQOL Group, 1996; WHOQOL Group, 1998). The WHOQOL‐BREF is a short version of the WHOQOL‐100 and is available in 19 different languages (WHOQOL Group, 1995, 1996; WHOQOL Group, 1998). The questionnaire includes four domains: physical, psychological, social relationships, and environment. Items are scored on a scale from 1 to 5, with a higher score indicating a higher quality of life. Two items are global indicators of quality of life and satisfaction with health; these are not included in the overall calculation of the domain scores (WHOQOL Group, 1996; WHOQOL Group, 1998). Domain scores are transformed on a scale ranging from 0 to 100 (Skevington et al., 2004). The WHOQOL‐BREF has shown to have good to excellent psychometric properties per reliability and validity across 23 countries (Skevington et al., 2004), and to be reliable, valid, and acceptable to use in samples of transgender women (Thompson et al., 2015).

#### 3.5.5 Additional survey content

Additional questions regarding gender identity and gender incongruence were included. The participants were asked the following questions: *How would you describe your gender identity?* With the following response options: ‘cisgender woman’ (i.e., female sex assigned at birth and identifies as woman), ‘transgender woman’ (i.e., male sex assigned at birth and identifies as woman), ‘cisgender man’ (i.e., male sex assigned at birth and identifies as man), ‘transgender man’ (i.e., female sex assigned at birth and identifies as man), ‘nonbinary’ (i.e., may identify as either female or male, both female and male, or a third separate gender, which often varies individually), or ‘other’. The participants were also asked: *What sex were you assigned at birth?* With the following response options: ‘female’, ‘male’, or ‘other’. Also, the participants were asked: *Have you ever experienced any incongruence between your sex‐assigned‐at‐birth and gender identity?* All questions regarding gender identity and gender incongruence were developed in collaboration with the centre for gender identity at Aalborg University Hospital, Denmark.

### 3.6 Ethics

The Danish health data authority approved data access. The study was reported to the research service (id‐number FSEID‐00006279), North Denmark region research register (id‐number F2022‐112), and to the North Denmark region committee on health research ethics, who stated that no ethical research approval was needed. Participants were informed that their participation was voluntary, their answers would be processed pseudo‐anonymously, and their participation would not affect any ongoing treatment they might be receiving.

### 3.7 Statistics

Descriptive analyses were carried out for all demographic variables, additional questions, and measures from the instruments described in section “Survey content”. The distributions of the continuous variables were assessed graphically. The number of answers was reported for all variables since this varied across the instruments due to partially completed questionnaires. Missing data were handled in the analyses using case wise deletion based on the availability of the data for individual variables. Dropout analyses were conducted to check for differences in sex and age between participants who returned the survey versus those who did not. Fischer's exact test was used to test for an association between sex and response. A two‐sample *t*‐test was used to estimate differences in age between responders and non‐responders.

The association between individuals identifying as transgender or gender‐diverse and those who identified as cisgender was assessed by creating a binary variable for gender identity. Cisgender males and females were coded as “cisgender” individuals, whereas individuals who identified as transgender female, transgender male, nonbinary, or other were coded as “transgender and gender‐diverse” individuals. Associations were tested by Fisher's exact test. The group of patients with an ED was compared to the non‐clinical comparison group on the results from the TCS, BUT‐A, and WHOQOL‐BREF instruments. To estimate the differences, a two‐sample *t*‐test was used. The assumptions of the two‐sample *t*‐test were checked graphically using residual plots. In cases where the assumptions could not be readily confirmed, bootstrapping with 500 replications was used to estimate standard errors to accommodate for potential violations.

A within group analysis of patients with EDs was conducted to estimate differences comparing individuals with a cisgender identity versus the transgender and gender‐diverse identity on the EDE‐Q, TCS, BUT‐A and WHOQOL‐BREF. The differences were estimated using the two‐sample *t*‐test. Like in the previous analyses, assumptions were checked graphically. In cases where assumptions could not be readily confirmed bootstrapping with 500 replications was used to estimate standard errors to accommodate for potential violations. Sensitivity analyses were carried out using the Wilcoxon rank‐sum test to assess the robustness of the statistical significance of the two‐sample *t*‐tests. A *p*‐value lower than 0.05 was considered statistically significant and each test was performed at a 95% confidence level. All analyses were performed in Stata 18 (StataCorp, 2023).

## 4 RESULTS

### 4.1 Descriptive analysis

In total, 568 patients with EDs (response rate = 41.6%) and 538 individuals from the non‐clinical comparison group (response rate = 19.7%) returned the survey and were included in the final sample (*N* = 1,106), corresponding to an overall response rate of 27.29%. No individual who was used twice in the matching process returned the survey.

**FIGURE 1:**
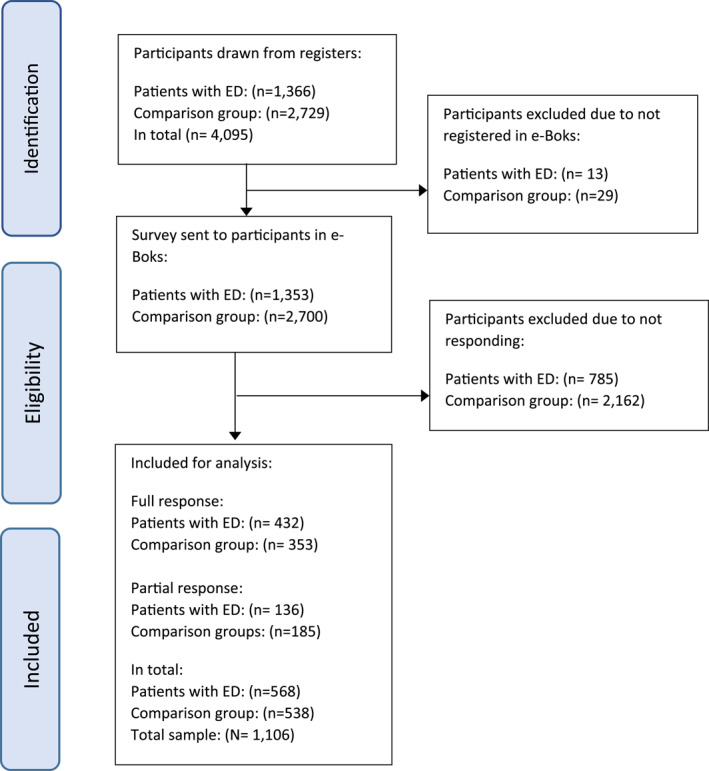
Flowchart. Flowchart showing study inclusion. A partial response was defined as accessing the survey and completing only the first questionnaire. Full response is defined as completing the last questionnaire, although missing data could occur throughout the response.

A flow chart illustrating the survey participation is shown in Figure 1. A dropout analysis showed no significant differences in age between those who returned the survey and those who did not. However, the distribution of registered sex between the responders and non‐responders was significantly different (*p* = 0.025; Fisher's exact test) among the non‐clinical comparison group, with 2,097 (95.71%) individuals registered as ‘female sex’ who did not return the survey compared to 526 (97.77%) who did. No significant association was found between registered sex and response rate among the patients with EDs.

Table 1 presents the descriptive characteristics and ED symptomatology of the study population. The median age of the two groups was comparable. As expected, patients with EDs had an elevated EDE‐Q on all subscales and the global scale score compared with the non‐clinical comparison group.

**TABLE 1:** Study characteristics and ED symptomatology.

	Patients with ED			Non‐clinical comparison group		
			n			n
Age	25.74	[21.34, 33.46]	568	26.18	[21.12, 33.30]	538
Socioeconomic status						
Employed	227	41.20%	551	194	37.96%	511
Enrolled in education	293	52.98%	553	397	77.54%	512
Other	152	26.76%	568	50	9.29%	538
Weight	59.25	(17.17)	434	72.02	(17.04)	397
Height	168.67	(6.83)	461	169.57	(6.83)	404
BMI	20.83	(5.93)	434	25.05	(5.70)	397
EDE‐Q						
Restraint	3.40	[1.60, 4.60]	474	0.80	[0.20, 2.40]	414
Eating concern	3.00	[1.80, 4.20]	469	0.20	[0.00, 1.00]	409
Weight concern	4.38	[3.25, 5.50]	470	1.88	[0.75, 3.38]	409
Shape concern	3.80	[2.60, 5.00]	470	1.80	[0.60, 3.00]	409
Global score	3.57	[2.55, 4.66]	469	1.20	[0.52, 2.33]	409

*Note*: Study characteristics and ED symptomatology. The data are represented in groups of three columns as either mean (sd) count, median [p25, p75] count, or frequency percent count. “n” is counted as non‐missing answers.

Abbreviation: EDE‐Q, Eating Disorder Examination Questionnaire.

Table 2 presents the prevalence of gender identity groups and the sex‐assigned‐at‐birth of the study population. In the group of patients with EDs, 4.50% (*n* = 20) identified as either transgender or gender‐diverse, versus 6.23% (*n* = 23) in the non‐clinical comparison group. These prevalences were estimated based on the sample size of (n) = 444 and (n) = 369, respectively. The difference between the groups was not statistically significant (*p*‐value = 0.276; Fisher's exact test). A total of 6.09% (*n* = 27) of the patients with EDs reported having experienced incongruence between their gender identity and sex‐assigned‐at‐birth, compared to 5.69% (*n* = 21) of the non‐clinical comparison group (*p*‐value = 0.882; Fisher's exact test).

**TABLE 2:** Comparison of patients with EDs and the non‐clinical comparison group of the prevalence of gender identity, sex‐assigned‐at‐birth, and on the Transgender Congruence Scale (TCS).

	Patients with ED			Non‐clinical comparison group			Two‐sample *t*‐test		
			n			n	Difference	95% CI	*p*‐value
Gender identity									
Cisgender individuals	424	95.50%	444	346	93.77%	369			
Transgender and gender diverse individuals	20	4.50%	444	23	6.23%	369			0.276^a^
Sex‐assigned‐at‐birth									
Female	428	96.40%	444	361	98.10%	368			
Male	16	3.60%	444	7	1.90%	368			0.202^a^
Selfreported experience of gender incongruence	27	6.09%	443	21	5.69%	369			0.882^a^
TCS									
Total scale	4.50	[3.92, 4.83]	423	4.92	[4.58, 5.00]	355	−0.38	[‐0.46, −0.29]	<0.001^b^
Gender identity acceptance	5.00	[4.33, 5.00]	431	5.00	[5.00, 5.00]	362	−0.25	[‐0.34, −0.16]	<0.001^b^
Appearance congruence	4.44	[3.78, 4.78]	427	4.89	[4.56, 5.00]	355	−0.42	[‐0.52, −0.32]	<0.001^b^

*Note*: Comparison of patients with EDs and the non‐clinical comparison group of the prevalence of gender identity, sex‐assigned‐at‐birth, and on the Transgender Congruence Scale (TCS). “n” is counted as non‐missing answers. The data in the first three columns are either frequency percent count or median [p25, p75] count.

^a^ Fischer's exact test (p‐value).

^b^ Bootstrapping with 500 replications was used to estimate standard error.

The majority identified as cisgender individuals with the largest group identifying as cisgender women, both for patients with EDs and in the non‐clinical comparison group (92.57%; *n* = 411) and 92.14% (*n* = 340), respectively. Thirteen (2.93%) cisgender men were identified among patients with EDs, compared to six (1.63%) in the non‐clinical comparison group. Five (1.13%) individuals identified as transgender men, and no individuals identified as transgender women among patients with EDs. In the non‐clinical comparison group, no individuals identified as transgender men. It was not possible to report numbers for transgender women in this group due to low numbers. Additionally, seven (1.58%) individuals identified as nonbinary among patients with EDs and 16 (4.34%) among individuals in the non‐clinical comparison group. Due to the low number of individuals identifying as ‘other gender identity’, we were not able to report specific frequencies for these groups. Furthermore, due to these small numbers, no statistical tests were conducted based on the gender identity subgroups. See Table 2 for a distribution of sex‐assigned‐at‐birth in the sample.

There was a statistically significant difference between the two groups on all subscales and the total scores on the TCS, with patients with EDs reporting the lowest level of gender congruence compared to the non‐clinical comparison group (Table 2).

The patients with EDs scored statistically significantly higher on all BUT‐A subscales and the global severity measure compared to the non‐clinical comparison group indicating more uneasiness with the body (Table 3). The patients with EDs reported statistically significant lower quality of life on all subscales of the WHOQOL‐BREF compared to the non‐clinical comparison group. Particularly, on the psychological domain, the patients with EDs scored −30.19 points lower than the non‐clinical comparison group (95% CI [−33.16, −27.22]; Table 3).

**TABLE 3:** Comparison between patients with EDs and the non‐clinical comparison group on the BUT‐A (Body Uneasiness Test) and WHOQOL‐BREF (WHO Quality of Life‐ BREF).

	Patients with ED			Non‐clinical comparison group			Two‐sample *t*‐test		
			n			n	Difference	95% CI	*p*‐value
BUT‐A									
Global severity measure	2.56	[1.88, 3.21]	417	0.76	[0.35, 1.56]	351	1.46	[1.32, 1.59]	<0.001^a^
Depersonalization	2.33	[1.50, 3.33]	443	0.33	[0.00, 1.00]	370	1.71	[1.57, 1.85]	<0.001^a^
Compulsive self‐monitoring	2.00	[1.20, 3.00]	444	0.60	[0.40, 1.40]	368	1.09	[0.95, 1.23]	<0.001^a^
Avoidance	1.67	[1.00, 2.50]	438	0.17	[0.00, 0.83]	366	1.22	[1.08, 1.36]	<0.001^a^
Body image concern	2.78	[1.89, 3.67]	436	1.06	[0.44, 2.00]	370	1.43	[1.27, 1.59]	<0.001^a^
Weight phobia	3.13	[2.25, 3.88]	438	1.00	[0.38, 2.12]	366	1.61	[1.46, 1.77]	<0.001^a^
WHOQOL ‐ BREF									
Environment scale	67.36	(17.28)	430	79.67	(15.82)	350	−12.30	[‐14.66, −9.95]	<0.001
Social relationships	54.08	(21.10)	430	67.95	(20.25)	350	−13.87	[‐16.80, −10.94]	<0.001
Psychological	36.48	(20.95)	429	66.67	(21.06)	350	−30.19	[‐33.16, −27.22]	<0.001
Physical	59.77	(18.07)	430	75.34	(15.46)	350	−15.57	[‐17.97, −13.18]	<0.001
Overall quality of life	3	[1, 5]	430	4	[1, 5]	350			
Overall perception of health	2	[1, 5]	426	4	[1, 5]	349			

*Note*: Comparison between patients with EDs and the non‐clinical comparison group on the BUT‐A (Body Uneasiness Test) and WHOQOL‐BREF (WHO Quality of Life‐ BREF). The data in the first three columns are either mean (sd) count, median [p25, p75], or mode [min, max] count. “n” is counted as non‐missing answers.

^a^ Bootstrapping with 500 replications was used to estimate standard error.

A within group analysis of cisgender individuals with EDs versus transgender and gender‐diverse individuals with EDs showed no statistically significant difference between the cisgender individuals and the transgender and gender‐diverse individuals on the global score or on any of the subscales of the EDE– *Q* (Table 4). As expected, the transgender and gender‐diverse individuals had statistically significant lower scores of gender identity acceptance and appearance congruence measured with the TCS, compared to the cisgender individuals. Also, regarding body uneasiness, measured by the BUT‐A, statistically significant differences were estimated on all subscales except for the compulsive self‐monitoring subscale, indicating that the transgender and gender‐diverse individuals with EDs reported more body dissatisfaction than the cisgender individuals with EDs. On the global severity measure a statistically significant difference was found of 0.68 (95% CI [0.21, 1.16]) between the transgender and gender‐diverse individuals with EDs versus the cisgender individuals with EDs. Finally, the transgender and gender‐diverse individuals with EDs reported a statistically significant lower quality of life on the WHOQOL‐BREF on all domains except the social relationship domain, compared to the cisgender individuals with EDs.

**TABLE 4:** Withing‐group analyses of cisgender individuals with EDs and transgender and gender‐diverse individuals with EDs.

	Cisgender individuals with ED			Transgender and gender diverse individuals with ED			Two‐sample *t*‐test		
			n			n	Difference	95% CI	*p*‐value
WHOQOL ‐ BREF									
Environment	67.80	(17.20)	410	59.45	(16.69)	19	−8.36	[‐16.28, −0.44]	0.039
Social relationships	54.42	(20.85)	410	46.05	(25.51)	19	−8.37	[‐18.09, 1.35]	0.091
Psychological	37.15	(20.89)	409	23.20	(18.07)	19	−13.95	[‐23.53, −4.36]	0.004
Physical	60.34	(17.99)	410	48.25	(16.26)	19	−12.10	[‐20.36, −3.83]	0.004
Overall quality of life	3	[2, 4]	410	2	[2, 3]	19			
Overall perception of health	3	[2, 4]	406	2	[2, 3]	19			
BUT‐A									
Global severity measure	2.50	[1.85, 3.15]	396	3.37	[2.59, 4.09]	18	0.68	[0.21, 1.16]	0.005
Depersonalization	2.33	[1.33, 3.17]	420	4.00	[2.50, 4.33]	19	0.98	[0.44, 1.53]	<0.001
Compulsive self‐monitoring	2.00	[1.20, 2.80]	421	2.40	[1.60, 3.20]	19	0.38	[‐0.14, 0.89]	0.151
Avoidance	1.67	[1.00, 2.50]	416	2.33	[1.83, 3.83]	18	0.65	[0.14, 1.16]	0.013
Body image concern	2.78	[1.89, 3.67]	414	3.67	[2.44, 4.78]	19	0.65	[0.08, 1.21]	0.025
Weight phobia	3.13	[2.25, 3.88]	416	3.69	[3.12, 4.38]	18	0.59	[0.04, 1.13]	0.035
TCS									
Total scale	4.50	[4.08, 4.83]	404	2.58	[2.25, 3.17]	18	−1.71	[‐2.04, −1.37]	<0.001
Gender identity acceptance	5.00	[4.33, 5.00]	412	2.67	[2.33, 3.33]	18	−1.89	[‐2.26, −1.53]	<0.001
Appearance congruence	4.44	[3.89, 4.89]	407	2.89	[2.22, 3.33]	19	−1.58	[‐1.99, −1.17]	<0.001
EDE‐Q									
Restraint	3.05	(1.75)	423	3.77	(1.77)	19	0.72	[‐0.09, 1.52]	0.081
Eating concern	2.92	(1.55)	420	3.12	(1.60)	19	0.20	[‐0.51, 0.91]	0.581
Weight concern	4.17	(1.48)	421	4.11	(1.90)	19	−0.06	[‐0.75, 0.63]	0.861
Shape concern	3.71	(1.54)	421	3.80	(1.86)	19	0.09	[‐0.62, 0.81]	0.797
Global score	3.46	(1.40)	420	3.70	(1.70)	19	0.24	[‐0.41, 0.89]	0.467

*Note*: Within‐group analyses of patients with EDs, comparing cisgender individuals versus transgender and gender‐diverse identity on the WHOQOL‐BREF (WHO Quality of Life‐ BREF), BUT‐A (Body Uneasiness Test), TCS (Transgender Congruence Scale) and EDE‐Q (Eating Disorder Examination Questionnaire). The data are represented in groups of three columns as either mean (sd) count or median [p25, p75] count. “n” is counted as non‐missing answers. Bootstrapping with 500 replications was used to estimate standard error for all the scales of the TCS.

## 5 DISCUSSION

This national, matched cross‐sectional study set out to estimate the prevalence of the transgender and gender‐diverse identity and to examine the levels of gender congruence, body dissatisfaction, and quality of life among individuals in treatment for EDs in Denmark. The findings showed that there was no significant difference in the prevalence of transgender and gender‐diverse individuals between patients with EDs and a non‐clinical comparison group. Thus, the results did not support our hypothesis. Nevertheless, the prevalence of transgender and gender‐diverse individuals found in this study somewhat corresponds with the results found in other studies, ranging between 2.2% and 7.1% in clinical samples of patients with ED (Chaphekar et al., 2022; Riddle et al., 2022, 2024). Furthermore, the prevalence of transgender and gender‐diverse individuals estimated in this study was high compared to results previously reported in the general population. Hilden et al. (2021) investigated the number of legal‐sex changes and gender identity‐related diagnoses in a Danish national cohort and found a prevalence of gender incongruence of 0.7% in the adult population. Additionally, the proportions of transgender and gender‐diverse individuals have ranged from 0.1% to 2% across populations in various countries worldwide (Goodman et al., 2019). Different reasons may explain the increased prevalence of transgender and gender‐diverse individuals in this study. One possible explanation could be the relatively young study population, with a median age below 26 years. Past research has suggested that the prevalence of transgender and gender‐diverse identities tends to be more frequent among younger individuals (Hilden et al., 2021; McKechnie et al., 2023; Nolan et al., 2019). Another explanation is the potential selection bias that may occur due to respondents' personal connection to the survey topics, potentially skewing the prevalence rates (Andrade, 2020). Conversely, respondents who could not relate to the topics might have avoided from responding, also influencing the prevalence rate. When assessing sensitive topics it impacts the number of individuals participating, the nonresponse rate, and how truthful answers given are (Tourangeau & Yan, 2007). Therefore, the results must be considered in view of the potentially higher prevalence associated with the young age of the study population and the impact of potential other biases.

Furthermore, we found a higher number of individuals identifying as transgender men compared to transgender women among patients with EDs; however, with five versus zero individuals, respectively, the numbers are very small and must be considered cautiously with no clear conclusion. Still, this finding corresponds to a previous systematic review and meta‐analyses which found that transgender men tend to have higher levels of EDs compared to transgender women (Rasmussen et al., 2023). Furthermore, our population was generated from a population of patients with EDs, resulting in a predominance of individuals assigned female‐sex‐at‐birth, which in turn increased the probability of including transgender men versus transgender women. Generally, an important aspect to consider when examining transgender and gender‐diverse individuals in clinical samples is that individuals with marginalised identities often experience a higher risk of facing barriers to ED treatment. It is more challenging for those individuals to receive an ED diagnosis and referral for ED treatment (Becker et al., 2003; Larson et al., 2021; Sonneville & Lipson, 2018). Studies have shown that transgender individuals face barriers to ED treatment, including discrimination, lack of knowledge and competencies among clinicians (Duffy et al., 2016; Hartman‐Munick et al., 2021). Thus, there may be a number of unreported cases that could potentially benefit from ED treatment but, for various reasons, may never be referred or receive treatment. The study also explored the level of gender congruence in patients with EDs. The results showed that patients with EDs did have a statistically significant lower level of gender congruence compared to the non‐clinical comparison group. However, although the results were statistically significant, it is important to mention that both groups reported high levels of gender congruence, which indicates that both patients with EDs and the non‐clinical comparison group, who primarily identified as cisgender, experienced high levels of gender congruence with their appearance and acceptance of gender identity. Moreover, when comparing our results with findings from previous studies measuring the gender congruence of (presumed) cisgender individuals, similar levels of gender congruence were estimated (Iliadis et al., 2020; Jones et al., 2019). Our findings showed that patients with EDs experienced more body dissatisfaction and a reduced quality of life compared to a non‐clinical comparison group. Especially, on the psychological domain on the of quality‐of‐life measure, patients with EDs had a lower score than the non‐clinical comparison group. This coheres with findings from a previous review, which showed that patients with EDs especially reported poorer quality of life on the mental component than in the general population (Baiano et al., 2014). Furthermore, regarding body dissatisfaction, the patients with EDs scored lower on all the subscales compared to the non‐clinical comparison group, indicating more dissatisfaction with the body. This, however, was expected as body dissatisfaction is one of the core symptoms of EDs and is associated with the development and maintenance of EDs (McLean & Paxton, 2019; Stice & Shaw, 2002) When comparing the transgender and gender‐diverse individuals with EDs to the cisgender individuals with EDs, no statistically significant difference was found in the ED symptomatology profile between the groups. Both groups reported high scores, particularly on the weight and shape concern scale. A potentially notable difference between the groups was only detected on the restraint scale. Our results are in contrast to two studies, which found higher ED symptomatology among treatment‐receiving transgender, gender diverse and sexual minority individuals compared to cisgender individuals with EDs (Coelho et al., 2021; Mensinger et al., 2020). However, they are in line with two other studies conducted in clinical samples of patients undergoing ED treatment, comparing ED severity between transgender and gender‐diverse individuals and cisgender individuals (Riddle et al., 2022, 2024). Both studies found no significant differences regarding EDE‐Q scores between the groups. The authors suggested that ED severity is similar across gender identities at the time of presentation to ED treatment, which might also be the case in this study.

Considering gender congruence, the transgender and gender‐diverse individuals with EDs expressed less gender congruence regarding their appearance and a lower acceptance of gender identity compared to the cisgender individuals with EDs. These findings are in line with previous results that found cisgender individuals to report significantly higher levels of gender congruence compared to transgender and nonbinary individuals (Jones et al., 2019). Furthermore, the transgender and gender‐diverse individuals with EDs reported more body dissatisfaction and a lower quality of life compared to the cisgender individuals with EDs. On the quality‐of‐life domains, the transgender and gender‐diverse individuals especially reported lower scores on the psychological and physical domains. These results were also found in a study where transgender individuals reported lower physical and mental quality of life compared to a control group (Valashany & Janghorbani, 2018). The transgender and gender‐diverse individuals with EDs had higher levels of body dissatisfaction than the cisgender individuals with EDs, also on aspects beyond weight phobia. Specifically, they reported higher levels of depersonalisation and avoidance, indicating more detachment and estrangement feelings towards the body and body image related avoidance behaviour (Cuzzolaro et al., 2006). The higher body dissatisfaction in transgender and gender‐diverse individuals with EDs in this study contrasts with previous studies that investigated body dissatisfaction among transgender individuals; they found transgender individuals to present with lower levels of body dissatisfaction compared to (presumed) cisgender individuals with EDs (Vocks et al., 2009; Witcomb et al., 2015). However, neither of these studies included transgender individuals diagnosed with an ED. As this study population is relatively young, factors such as age and gender‐affirming treatment are relevant to consider regarding body dissatisfaction and quality of life. Studies have shown that older transgender individuals tend to have better body image and psychological well‐being, suggesting age as a protective factor, and that gender‐affirming treatment may improve the quality of life and body dissatisfaction (Meneguzzo et al., 2024; Nobili et al., 2018; van Leerdam et al., 2023). Younger transgender and gender‐diverse individuals may be in the initial process of their transition, which potentially may impact their quality of life and body dissatisfaction. Hence, transgender and gender‐diverse individuals with an ED might be a specific group within this population with severely marked body image concerns and low quality of life. Overall, our transgender, gender‐diverse and cisgender participants with EDs did not differ significantly regarding ED symptomatology. However, the results do point towards transgender and gender‐diverse individuals with an ED experiencing worse quality of life and more body dissatisfaction when compared to cisgender individuals with an ED. Thus, this implies the presence of additional considerations regarding body dissatisfaction and quality of life for transgender and gender‐diverse individuals with EDs, which are important to be aware of in the clinical treatment of EDs, particularly in younger individuals. Furthermore, clinicians may be aware of whether the individuals are receiving or desiring gender‐affirming treatment, as transitioning may generate concerns about “passing” potentially impacting both body dissatisfaction and ED‐related behaviours (Jones et al., 2016; Riddle et al., 2022; Roberts et al., 2021). This emphasises the importance of considering the unique experiences and challenges faced by transgender and gender‐diverse individuals in the context of ED treatment.

## 6 STRENGTHS AND LIMITATIONS

This nationwide, matched cross‐sectional study included both registry and survey data. The use of nationwide registry data, enabling the inclusion of all patients registered with contact related to an ED in Denmark strengthens our ability to generalise our findings within this population. However, it is important to acknowledge that the participants included represent a selectively chosen patient group, which imposes a limitation on our ability to generalise our findings to individuals with less severe forms of EDs or those who have not sought treatment (Schmidt et al., 2015). Nevertheless, the organisation of a free health care system in Denmark, providing centralised treatment for all cases of EDs, leads to the inclusion of most clinical cases in the registries. Furthermore, through the utilization of three national Danish registries, we reduced the potential selection bias which could otherwise be introduced from selective inclusion of specific hospitals (Schmidt et al., 2015). Still, this does not preclude the presence of selection bias as the topic of the survey may have attracted or refrained individuals from responding, potentially influencing the prevalence. Both selection bias and low response rates can be anticipated from survey studies, which is why the study provides a cautious estimate of the prevalence of transgender and gender‐diverse individuals within an ED population (Andrade, 2020; Sammut et al., 2021). Furthermore, another bias potentially impacting the higher number of transgender men found in this study was due to the fact that we almost exclusively included (and received responses from) individuals registered as female sex. However, this study is an important contribution to assessing the prevalence of transgender and gender‐diverse individuals in ED populations. Although, due to its descriptive setup, the study can only provide indications of the prevalence. Further studies are necessary to corroborate our findings of the prevalence of transgender and gender‐diverse individuals in clinical samples of ED populations.

## 7 CONCLUSION

In this study, the prevalence of transgender and gender‐diverse individuals was estimated at 4.50% among patients with eating disorders. Overall, we did not find a higher prevalence of individuals identifying as transgender and gender‐diverse among patients with eating disorders compared to a matched non‐clinical comparison group. However, the results suggest that transgender and gender‐diverse individuals with eating disorders experience a lower quality of life and more body dissatisfaction compared to cisgender individuals with eating disorders. These findings contribute to important groundwork for further investigations of clinical treatments of eating disorders, which may enable health care professionals to better address the needs of transgender and gender‐diverse individuals with eating disorders.

## CONFLICT OF INTEREST STATEMENT

The authors declare no conflicts of interest.

## Data Availability

Research data are not shared.
